# Phonation differentiation by non-contact laryngeal magnetomyography

**DOI:** 10.1038/s41598-025-02956-2

**Published:** 2025-05-29

**Authors:** Justus Marquetand, Nima Noury, Hongyu Lu, Haodi Yang, Chrystina Montuori Sorrentino, Lukas Rüttiger, Marlies Knipper, Christoph Braun, Hubert Löwenheim, Johannes von Fraunberg, Anke Tropitzsch, Markus Siegel, Stephan Wolpert

**Affiliations:** 1 Department of Neural Dynamics and Magnetoencephalography, Hertie Institute for Clinical Brain Research, University of Tübingen, Otfried-Müller-Str.47, 72076 Tübingen, Germany; 2 MEG-Center, University of Tübingen, Otfried-Müller-Str.47, 72076 Tübingen, Germany; 3 Center for Integrative Neuroscience, University of Tübingen, Otfried-Müller-Str.47, 72076 Tübingen, Germany; 4 Institute for Modelling and Simulation of Biomechanical Systems, University of Stuttgart, Stuttgart, Germany; 5 Department of Otolaryngology, Head and Neck Surgery, University of Tübingen, Tübingen, Germany

**Keywords:** Larynx, Voice, Quantum sensor, Neurophysiology, Singer, Speech, Translational research, Physiology, Neurophysiology

## Abstract

Phonation is important for our daily communication and requires the activation of internal and external laryngeal muscles, which can be recorded by electromyography (EMG) using surface or needle electrodes. Here we present a new noncontact method, laryngeal magnetomyography. As a proof-of-concept, we investigated the feasibility of differentiating various vocalization conditions using laryngeal MMG in two healthy subjects using optically pumped magnetometers (OPM). We recorded magnetic muscle activity of the larynx and neighboring cervical muscles using a 3 × 5 array of OPMs. Subjects vocalized an /a/ in three different conditions: loud high pitch, loud low pitch, and soft high pitch, in 90 s blocks. After removing cardiac artifacts, MMG signals were in the range of 1.5 pT with significant amplitude differences between conditions. In both subjects, Linear Discriminant Analysis (LDA) was able to significantly classify vocalization conditions based on the spatial pattern of MMG activities. In sum, we show that laryngeal MMG allows contactless differentiation of phonations based on myomagnetic signals. Our results set the stage for future studies to explore this method for clinical diagnostics and therapy. Functional, contactless muscle recordings during vocalization enable new applications for miniaturized quantum sensors, e.g. in linguistic studies and speech rehabilitation.

## Introduction

Human phonation is of paramount importance for communication, expression, and delivery of everyday speech. The fine coordination of laryngeal muscles determines the quality of the voice and its characteristics^1,2^. Central to phonation is the larynx, a complex structure consisting of several fine external and internal muscles and nine cartilages^3^. Detecting the activity of the laryngeal muscles is important for diagnosing and monitoring laryngeal health, whether indirectly through listening, visual inspection (laryngoscopy) during phonation, or by measuring electrical activity using electromyography (EMG)^2^.

To date, the best-established method is conventional EMG using needle electrodes, which must be inserted into the muscles and allow measurement of action potentials from motor units of deeper muscles^4^. However, this invasive and potentially uncomfortable method requires considerable experience and has limitations for patients with bilateral dysfunction or anticoagulants who may experience swelling or bleeding in the larynx^2,5^.

Surface EMG (sEMG) is the standard non-invasive alternative to needle EMG. It captures myoelectric signals from muscles or muscle groups closer to the skin surface^4^. Although several studies^6–10^ have demonstrated the ability to measure laryngeal activity for different phonations using sEMG, as in other areas of clinical neurophysiology, sEMG is not established in daily routine due to various limitations such as application time, complex non-automated post-processing, and most importantly, because it lacks specificity to laryngeal muscles, making it less useful for diagnostics and treatment response monitoring^11,12^.

An elegant solution to overcome the need for electrode placement is to measure the magnetic counterpart of EMG, i.e. magnetomyography (MMG). MMG measures the magnetic fields that are produced by the secondary intracellular and extracellular currents that result from primary transmembrane ionic currents along the muscle fibers^13,14^. Consequently, EMG and MMG measure different byproducts of the same muscle electrical activity. Furthermore, while EMG records scalar voltage signals, MMG records three dimensional vectorial magnetic signals according to the right-hand-rule of electromagnetism, which may offer advantages in signal decomposition^15^.

The idea that MMG could be used as a noncontact clinical neurophysiological modality was first proposed in early studies in 1972, when Cohen et al.^16^ measured biomagnetic activity in vivo using a superconducting quantum interference device (SQUID). However, further MMG studies have been sparse, likely due to the technical limitations associated with SQUID sensors and their high cost. In particular, the need for cryogenic cooling to −269 °C (4.15 Kelvin) and the lack of spatial flexibility made it difficult to measure individual anatomical situations. These limitations of SQUIDs, i.e. lack of spatial flexibility and the need for cryogenic cooling, have been overcome by recent miniaturized quantum sensors such as the optically pumped magnetometers (OPMs). In short, OPMs measure magnetic fields by utilizing the interaction between polarized light and alkali metal atoms, such as rubidium or cesium, in a vapor cell. A polarized laser optically pumps these atoms, aligning their spins along the laser direction. When exposed to an external magnetic field, such as those from bioelectromagnetic activity, the atomic spins undergo Larmor precession. This precession alters their interaction with the laser light, causing the vapor to absorb some of it. By measuring this absorption, the magnetic field strength can be detected and quantified^17^. OPMs offer the advantage of recording with an individually arranged sensor setup and at a much lower cost compared to SQUIDs, thus providing ideal opportunities to explore the applicability of MMG for clinical studies. A potential new field for MMG is the establishment of a new contactless neurophysiological modality for neuromuscular assessment of the laryngeal muscles. Here, we demonstrate, for the first time, the feasibility of such an approach. We performed a proof-of-principle study in two healthy subjects and investigated laryngeal MMG during different pitches of loud and soft phonation. Specifically, we hypothesized that it is possible to discriminate between different phonation conditions at the level of a single subject.

## Material and methods

### Participants

Two healthy subjects (2 males, ages 28 and 36; body-mass-index: 21 and 22 kg/m^2^) participated in the study. Both were authors (JF, JM) of this study and gave informed consent to participate and publish their respective data. A pilot experiment was conducted over two days in September 2023 at the Department of Otolaryngology, Head, and Neck Surgery of the University Hospital of Tübingen. After data analysis, both subjects participated in a second experiment in March 2024 at the MEG-Center Tübingen, the results of which are reported here. All experiments were conducted according to the standards of the World Medical Association. This study was registered and approved by the ethics committee of the University of Tuebingen. All subjects consented to participate in the study and consented to publication of anonymized data.

### Measurement procedures

**Fig. 1:**
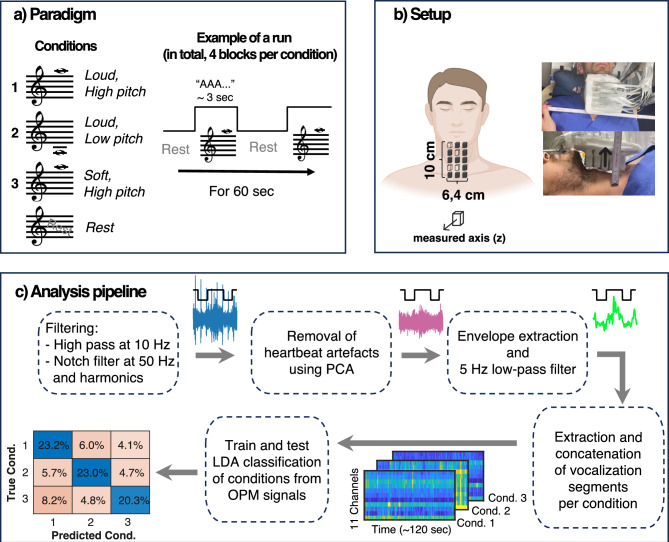
Overview of the study paradigm, setup, and data analysis. **a**) The experiment consisted of three vocalization conditions: loud high pitch, loud low pitch, and soft high pitch. Subjects maintained a pitch in approximately 3 s intervals. Between these singing intervals, subjects could rest. The experiment was conducted in 60 s blocks (12 blocks in total), and, in each block, subjects performed one of the three singing conditions. Note that the definitions of high, low, loud, and soft were subjectively determined by each participant, and the musical notes shown in this figure are for illustration only and do not correspond to specific vocal frequencies. **b**) Larynx activity was recorded using 15 OPMs, 4 of which showed malfunctions. This resulted in 11 working OPMs (filled cubes in the schematic plot). **c**) Summary of data analysis. In each block, recorded signals were first filtered (high pass at 10 Hz and notched at 50 Hz and its harmonics up to 250 Hz). Then, the heartbeat artifact was removed using PCA. Next, signal envelopes were extracted using the Hilbert transform. We low-pass filtered these envelopes at 5 Hz. Next, we extracted intervals in which subjects sang based on the trigger signal (black rectangular trace) and concatenated all segments of the four blocks of each condition to construct condition matrices. Finally, we trained and tested an LDA classifier on these three matrices.

Both participants repetitively vocalized the phonetic/a/in 60 s blocks (Fig. 1, paradigm). Each block was pseudorandomly assigned to one of three vocalization conditions: loud high-pitch, loud low-pitch, and soft high-pitch (although a soft low-pitch condition was included in the above-mentioned pilot experiment, we excluded it from the main experiment to shorten its duration). To prevent temporal biases, we randomly assigned each successive set of three blocks to conditions one, two, and three. In other words, we made sure that each successive set of three blocks contained all three conditions. This procedure was repeated four times, resulting in a total of 12 blocks. The realization of what was considered high, low, loud, and soft was subjectively decided by each participant. Within each block, participants were asked to vocalize repetitively for approximately 3 s and rest briefly between successive vocalization intervals. The entire procedure was self-paced without external cues. During vocalizations, subjects pressed a custom-made non-magnetic button, whose signal was recorded using the same acquisition device as for the MMG-Signal and was used later as a trigger to define vocalization intervals. For conditions 1, 2 and 3, this procedure resulted in 58, 60, and 59 vocalization pieces, respectively in subject 1, and 40, 39, and 47 vocalization pieces, respectively in subject 2. The average length of the vocalization intervals was 2.46 ± 0.61, 2.53 ± 0.51, and 2.53 ± 0.55 s (mean ± std) for subject 1, and 4.47 ± 0.67, 4.68 ± 0.75, and 3.87 ± 0.75 s for subject 2, corresponding to conditions 1, 2 and 3, respectively. Subjects were free to speak or swallow during the rest intervals. Therefore, we did not include these intervals in our analysis.

### Measurement setup

MMG was measured using 15 Fieldline v2 OPMs (Fieldline, Boulder, CO, USA) inside a magnetically shielded room (Ak3B in Tübingen, designed by Vacuumschmelze, Hanau, Germany). OPMs were aligned in a 5 × 3 array with 0.5 cm spacing between them, resulting in an area of 10 × 6.4 cm (Fig. 1, setup) that was mechanically decoupled from the subject. The array consisted of a custom-made polystyrene holder that was manually prepared for an array design. Due to the dense array and the material properties of polystyrene, the OPMs could not move and maintained their position. We did not measure the exact distance between each OPM and the skin, but aimed to keep the distance between sensors and skin below 1.5 cm. When considering the distance, it should be noted that during vocalization, the relative distance between the sensors and the source, i.e. the larynx, varies due to movement of the larynx. This circumstance is fundamentally different from brain recordings, where the position of the sensors relative to the signal source theoretically remains constant because the signal source does not move.

### Data acquisition

OPMs were recorded on the Z-axis (facing the ventral side of the laryngeal and cervical muscles, Fig. 1) in closed-loop mode. The trigger button press generated a simultaneously recorded analog rectangular signal for the duration of the button press. Both signals (OPM and button press) were recorded at 1000 Hz sampling rate.

### Data analysis

Data analysis was performed in MATLAB (Version R2021b, MathWorks Inc., Natick, Massachusetts, United States) using custom scripts and the open-source toolbox FieldTrip^18^. Raw signals were notch filtered at 50 Hz and harmonics up to 250 Hz, and high-pass filtered at 10 Hz (both filters were forward-reverse third order Butterworth filters). Then, in each 60 s block, we applied principal component analysis (PCA) and removed the first principal component (PC) to suppress heartbeat artifacts. Next, we estimated the signal envelopes using the Hilbert transform, low-passed the envelope at 5 Hz, and, using the trigger signal, segmented the envelopes into intervals in which the subjects vocalized (pay attention that the envelope of a 10 Hz high-passed signal could still contain changes slower or faster than 10 Hz). We utilized this preprocessed data for all the subsequent analyses.

After preprocessing, for each subject, we combined all vocalization segments assigned to a single condition to generate three matrices corresponding to the three conditions, and compared the medians of the distributions of envelopes between conditions. For each condition and subject, we created a single distribution of the decimal logarithm of all the envelopes across all time points and all channels. This gave us three distributions for each subject. We then compared the median of these distributions in pairwise permutation tests, separately for each subject. We took each two distributions and randomly shuffled their samples to generate two random distributions. We then calculated the difference between the medians of these two random distributions. We repeated this 1000 times to generate the null distribution of random median differences and compared the true median difference to this null-distribution to obtain a p-value (2-sided).

Next, separately for each subject and using the three above-mentioned matrices that were obtained by concatenating all vocalization segments of each condition, we trained and tested a tenfold linear discriminant analysis (LDA) classifier to discriminate the three conditions. To avoid a bias due to differences in sample size, we stratified the number of samples to that of the condition with the fewest samples. We tested the performance of this classifier in a permutation test, where we randomly permuted all condition labels to assign a random label to each segment, trained and tested a tenfold LDA on this permuted dataset, and calculated its F1. We repeated this 1000 times to create a distribution of random F1 values and compared the F1 of the original classifier to this null distribution to obtain a p-value.

For calculating the Z-scored envelope values per channel and condition (Fig. 3), we only included preprocessed vocalization segments that were longer than two seconds. Furthermore, to eliminate potential biases caused by differences in vocalization length across conditions, we extracted only the first two seconds of each selected piece. The second condition of the second subject had 39 vocalization pieces longer than two seconds, which was the lowest count across all conditions and subjects. To ensure balanced data, we randomly selected 39 pieces from each condition for each subject and concatenated them. This resulted in a vector containing 39 × 2 × 1000 = 78,000 samples per channel, condition, and subject. We then normalized each channel’s data across the three conditions within each subject by concatenating the three vectors per channel and computing the Z-score for each sample.

To compare the medians of the above-mentioned Z-scored envelope values across different conditions, we performed a permutation test for each channel, separately for each subject. As our test statistic, we used the sum of the squared differences of the medians across the three conditions. To approximate the null distribution, we randomly shuffled the condition labels and recalculated the test statistic 1000 times. The p-value was then estimated by comparing the observed test statistic with this null distribution.

Using the Z-scored envelope values, we also performed an LDA classification across subjects. The classifier was trained on all Z-scored values from one subject and tested on the Z-scored values of the other (i.e., without k-folding). To obtain a p-value, we estimated the null distribution by randomly shuffling the condition labels in the test dataset and repeating the classification 1000 times. The same Z-scored values were also used to train an LDA classifier on the combined data from both subjects. To prevent biases in each fold toward one subject, we ensured that each fold contained an equal amount of data from both subjects.

## Results

**Fig. 2:**
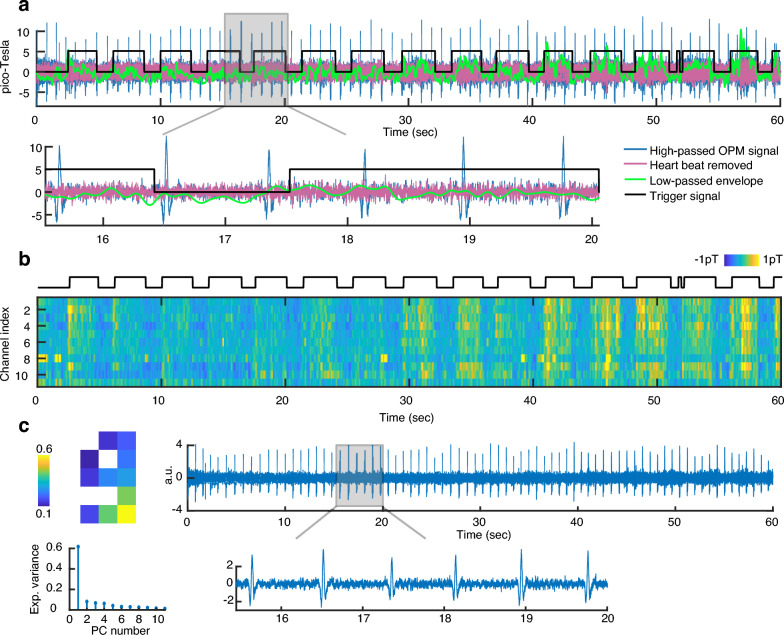
**a**) Time domain signal of an OPM sensor during an experimental block assigned to condition 1 (loud high pitch) from the first subject. The black rectangular waveform depicts the trigger signal that marks singing (high values) and silence (low values) intervals. The blue line shows the 10 Hz high-pass filtered signal containing strong heartbeat artifacts. The Magenta line shows the same signal after removing the first PC, which leads to a strong suppression of heartbeat artifacts. The green line shows the low-pass filtered (5 Hz) envelope of the artifact free signals. For visualization purposes, the low passed envelope is mean-removed and magnified by a factor of 10. **b**) Low-pass filtered (5 Hz) and mean-subtracted envelopes of all channels for the same block as (a). The black waveform depicts the trigger signal. **c**) PCA analysis of the same block. The top left plot depicts PCA loadings of the first PC, which are mapped to the sensor positions. The time domain activity of this PC (right plots) shows that this PC mainly reflects heartbeat artifacts. The OPM positioned at the bottom left corner, which was closest to the heart, had the highest loading for the first PC. The bottom left plot shows the signal variance explained by different PCs. The first PC explained about 60% of the variance in this block.

We observed strong heartbeat artifacts in high-pass filtered signals (Fig. 2a, blue curve). To remove these artifacts, we applied PCA (principal component analysis) to the raw signals of each block. Due to the strength of the heartbeat artifacts, the first PC captured this artifact (explained variance of about 60%, Fig. 2c) and its removal resulted in heartbeat free signals (Fig. 2a, magenta curve).

**Fig. 3:**
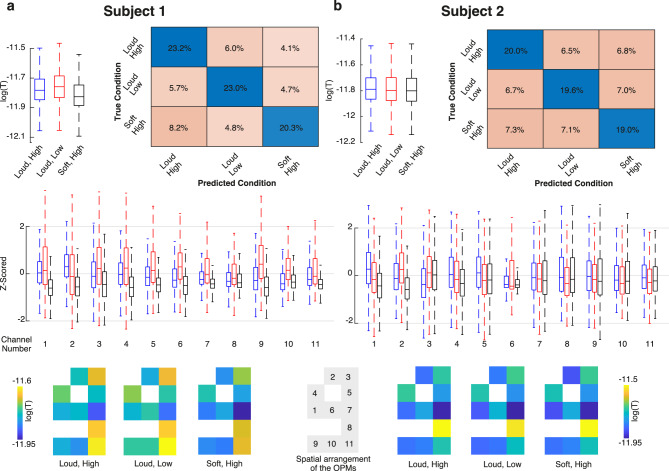
Classification results. **a**) Results of the first subject. For each condition, the top left box plot shows the distribution of envelope values of all OPMs across all time points. The middle line, box bottom and top edges and whiskers indicate the median, the first and third quartiles, and the minimum and maximum, respectively. Using a permutation test, we compared the median values of these distributions, and all pairwise tests led to significant differences with p < 0.001. The boxplot in the middle illustrates the distribution of Z-scored envelope values per channel and condition. For each channel, the left, middle, and right boxes represent the loud high, loud low, and soft high conditions, respectively (Z-scoring was applied across conditions for each channel separately). All channels exhibited significant differences between the medians of the Z-scored envelope distributions (all p-values < 0.001). The top right depicts the confusion chart (total normalized), resulting from a tenfold cross-validated LDA classifier (F1 = 0.66, p < 0.001). The bottom row shows the mean envelope values of the OPM array for each condition. Despite the subtle differences, the LDA classifier was able to distinguish the three phonation conditions from each other. **b**) Results of the second subject. All statistical tests related to the box plots led to significant differences with p < 0.001. Classification using LDA resulted in F1 = 0.59, p < 0.001.

We first investigated whether the strength of the preprocessed and artifact-removed MMG signals from the different conditions showed any significant differences. We estimated signal strength by obtaining the signal envelope of each channel using the Hilbert transform and low-pass filtering (Fig. 2a, green curve, Data analysis). We then created a population of signal strengths for each condition by concatenating all data points of all channels and performed pairwise permutation tests between conditions (Data analysis). Indeed, all pairwise tests resulted in significant differences (all p < 0.001, top left box plots in Figs. 3a-b, population medians for conditions 1 to 3 were 1.651 pT, 1.743 pT, and 1.492 pT for subject 1 and 1.628 pT, 1.594 pT, and 1.583 pT for subject 2, respectively). To investigate vocalization effects on the MMG signal of individual OPM sensors, we plotted and compared the z-scored envelopes across vocalization conditions for each OPM channel (Fig. 3, middle boxplots). All channels exhibited significant differences between the medians of the Z-scored envelope distributions (all p < 0.001).

Next, we investigated whether the different conditions showed unique activity patterns across OPMs. In other words, we investigated whether it was possible to differentiate vocalizations by analyzing the spatial activity patterns of OPM signals across sensors (Fig. 3, bottom plots). Indeed, although only 11 uniaxial OPMs were available, the LDA classifier could significantly discriminate the three phonation conditions from each other (confusion matrices in Figs. 3a-b, tenfold cross-validated LDA, F1 = 0.66 and 0.59, in subjects 1 and 2, respectively; both p < 0.001). The accuracy of the LDA classifiers was 66% and 59% in subjects 1 and 2, respectively.

Do the activity patterns of subjects share similar features? To test this, we performed an across-subject LDA classification, i.e., training the classifier on one subject and testing it on the other. In both cases, the classifier achieved an above-chance accuracy (38% and 36% for training on the first subject and testing on the second, and vice versa). Despite the relatively low accuracies, permutation tests confirmed that both classifications were statistically significant (p < 0.001). These results suggest that the activity patterns of the subjects share some common features. This similarity is evident in the distribution of signal strengths across different conditions. For instance, in both subjects, many channels showed stronger activity in the loud high-pitch condition compared to the soft high-pitch condition (Fig. 3, middle boxplots). However, the amplitude change induced by the loud low-pitch condition varied inconsistently between the two subjects (Fig. 3, middle boxplots). To further investigate this, we trained a new LDA classifier on the combined data from both subjects. In fact, this classifier made more use of the similarities between the two subjects and achieved a higher accuracy (57%) compared to the across-subject classification (within class accuracies were 60.2%, 44.7% and 65%, respectively, for loud high-pitch, soft high-pitch, and loud low-pitch). In sum, these findings suggest that while individual differences exist, there are also shared features in the activity patterns of different subjects.

In sum, our MMG recordings showed phonation specific properties. More specifically, by looking at the spatial activity pattern of OPMs, we were able to significantly differentiate vocalization conditions.

## Discussion

This proof-of-principle study with healthy non-singing participants performing different/a/pitches is, to the best of our knowledge, the first study to investigate laryngeal biomagnetic muscle activity. Despite limitations of the setup (see below), which may affect the performance of OPM to measure laryngeal MMG, we were able to demonstrate that it is possible to discriminate between different vocalization conditions at the single-subject level and provide first reference amplitudes for future studies of laryngeal MMG. Our results provide first evidence that laryngeal MMG may offer a new noncontact modality for diagnostics, monitoring, and therapy (e.g. neurofeedback).

In general, there is a need for improved methods for voice diagnostics. One of the most common diagnoses in patients with voice disorders is muscle tension dysphonia (MTD). Although several new methods have been proposed, there is currently no established objective tool to assess laryngeal function in these patients^19–22^. Although more than 30 years ago the first sEMG studies showed some positive results with a limited number of patients, there is still only moderate level of evidence for the diagnostic value of sEMG in identifying MTD^6,7,10,12,20^. The limited number of studies on sEMG for voice diagnostics may stem from practical and diagnostic challenges, including the time-consuming electrode application process (such as skin preparation) and the difficulties of post-processing a modality with inherently low spatial resolution^23^. One approach to partially overcome low spatial resolution is to use high-density laryngeal sEMG, which uses a greater number of skin electrodes^24,25^. However, practical limitations persist due to the requirement for direct electrode-to-skin contact. Here, MMG could serve as a new modality to overcome these practical limitations, as subjects only need to position their neck beneath the sensors with no preparation time required.

Based on the feasibility of laryngeal MMG that we have demonstrated here, what specific benefits need to be systematically investigated in the future? First, a non-contact examination could allow repetitive monitoring of laryngeal muscle disorders, i.e. MMG could be used as a new neuromuscular monitoring modality. Second, using an array design with a high density of sensors could enable functional mapping of neuromuscular activity as a new type of imaging. In this context, it is particularly advantageous that the mapping field generated by the electrical muscle activity is spatially selective (according to the so-called right-hand rule) and predominantly oriented orthogonal to the direction of the muscle fiber. This is both an advantage and a disadvantage because, on the one hand, additional spatial information is potentially obtained, but this would also need to be taken into account by co-registration of the moving signal source relative to the sensors (i.e., moving muscles during phonation). The above-mentioned potential development of using multiple sensors for MMG could enable contactless functional imaging, which could be used for diagnostics, monitoring, and therapy (e.g., using a biofeedback approach in laryngeal palsies). Third, MMG can theoretically measure deeper signal sources^15^, which may be particularly useful for clinical applications where surface EMG may not be able to record deeper laryngeal muscle activity, thus necessitating the use of invasive needle EMG. Our proof-of-principle study sets the stage to systematically investigate all these opportunities and challenges of laryngeal MMG.

### Limitations and future directions

Our proof-of-principle study has several limitations that should be addressed in future studies. For example, it would be ideal to simultaneously record source position, distance and orientation relative to OPM sensors. It is important to note that during phonation, the relative distance between the sensors and the larynx might vary due to laryngeal movement. Future studies should systematically investigate the impact of sensor-to-skin distance on MMG measurements to enhance experimental replication and result interpretation. Another valuable addition for future studies would be simultaneous MMG and sound recordings. This would allow a direct correlation between the vocalized sound and MMG signals. Another shortcoming of the current study is the lack of simultaneous EMG recordings. Simultaneous high-density EMG recordings would enable a direct quantitative comparison between laryngeal EMG and MMG. Such simultaneous recordings are necessary to investigate the amount of non-redundant information MMG provides relative to EMG. Moreover, it remains an open question whether MMG shares the same lack of specificity as EMG in identifying laryngeal muscles, especially since both techniques measure compound neuromuscular activity, likely capturing signals from surrounding muscles in addition to the laryngeal muscles.

Unlike scalar electrical potentials using EMG sensors, OPMs and other magnetic sensors record magnetic field vectors. The direction of each muscle’s magnetic field depends on the exact shape and orientation of the muscle. Therefore, uniaxial sensors, as used in the present study, only measure the part of the magnetic field that aligns with the measurement axis. Future studies may employ triaxial sensors to generate comprehensive functional maps of laryngeal muscles. Furthermore, the current bandwidth of the utilized OPM in this study is limited to 350 Hz, whereas muscle signals contain information above that. Further studies should address if and how this bandwidth limitation might be a relevant factor for MMG (e.g., comparing OPM with SQUID, which has a higher bandwidth).

Our results show that various vocalization conditions can be classified using MMG. However, in this study, we did not aim to determine the limits of such classification. Future research could explore this further and enhance classification accuracy by modifying different aspects of our approach. First, a better-controlled experimental setup that further restricts head and neck movements could improve classifier performance. Any movement alters the activity mapping observed by the OPMs, thereby deteriorating classification accuracy. Additionally, given the proof-of-concept nature of our study, we did not optimize preprocessing settings or explore features beyond the signal envelope. As a result, our findings are likely far from optimal, even with the current dataset. Using alternative preprocessing settings or incorporating additional signal features, such as time–frequency features or features extracted using PCA or ICA, could enhance performance. Finally, alternative machine learning approaches may further improve classification accuracy. For instance, a recent study from our group demonstrated that deep learning models could decode single-finger movements using MMG with an accuracy of 89%^26^. Such an approach is likely to be beneficial for this application as well.

The subjective nature of high/low and loud/soft determinations may introduce variability and affect reproducibility. Additionally, since this study did not systematically assess whether participants used thyroarytenoid- or cricothyroid-dominant registration (chest voice or falsetto), the potential influence of vocal registration on the results remains unclear. Furthermore, our study did not perform vocal registration as it was not within the scope of our proof-of-principle design. Future studies should incorporate objective measures to account for these factors and improve consistency.

Regarding future directions of laryngeal MMG, this new modality could be particularly useful for clinical neurophysiology, i.e., in studies with patients with laryngeal disorders (e.g., unilateral vagal nerve palsy after thyroidectomy). Here, being contactless could not only be of a pragmatic advantage for diagnostics, monitoring or therapy but also foster neurophysiological research of the larynx.

## Data Availability

The anonymized data of both participants are stored locally, and any raw data can be made available upon reasonable request to the corresponding author (JM).

## Abbreviations

OPM: Optically Pumped Magnetometer
EMG: Electromyography
sEMG: Surface Electromyography
MMG: Magnetomyography
OPM-MMG: Optically Pumped Magnetometer Magnetomyography
MEG: Magnetoencephalography
SQUID: Superconducting Quantum Interference Device
LDA: Linear Discriminant Analysis
